# Microbial degradation of phenol and derivatives using environmental isolates from industrial waste sources

**DOI:** 10.1007/s10482-026-02259-0

**Published:** 2026-02-05

**Authors:** Selin Saricayir, Tayyibe Alpay, Bike Pashayeva, Ayhan Ezdesir, Guven Ozdemir

**Affiliations:** 1 Department of Biology, Section of Basic and Industrial Microbiology, Ege University, Izmir, Turkey; 2 SOCAR Turkey R&D and Innovation Co., Refinery & Petrochemical Business Unit, Izmir, Turkey

**Keywords:** Phenol degradation, Refinery wastewater, Bioremediation, *Microbacterium arabinogalactanolyticum*, *Brevundimonas diminuta*

## Abstract

This study investigates the microbial degradation of phenolic compounds using environmental bacterial isolates obtained from refinery wastewater and petroleum-contaminated soil. Phenolic pollutants are highly toxic and persistent, posing significant challenges for biological wastewater treatment systems. To address this issue, microorganisms were enriched under increasing phenolic loads using Bushnell Haas Yeast (BHY) medium supplemented with phenol and mixed phenolic derivatives as the sole carbon source. Through adaptive passaging, two phenol-tolerant isolates were obtained and identified by 16S rRNA sequencing as *Microbacterium arabinogalactanolyticum* (PKN7) and *Brevundimonas diminuta* (VGT4). Time-resolved HPLC analyses demonstrated that both isolates completely degraded phenol within 120 h in BHY medium containing 20 mg/L phenol and 30 mg/L mixed phenolic compounds. While the strains exhibited only partial degradation of chlorophenols and cresols, consortium experiments showed *enhanced performance in the mixed culture*: the mixed culture achieved complete degradation of 2,4-dinitrophenol within 12 h and complete phenol removal within 60 h, while removing 73–78% of the remaining phenolic derivatives. These results confirm that cooperative metabolic interactions substantially enhance degradation performance under mixed-pollutant conditions. Overall, this study identifies *M. arabinogalactanolyticum* and *B. diminuta* as promising non-model phenol degraders, particularly when applied as a defined microbial consortium. Their combined activity highlights the potential for bioaugmentation-based strategies in industrial wastewater treatment systems. Further pilot-scale studies using real refinery effluents are needed to evaluate long-term stability and field applicability.

## Introduction

Environmental pollution remains one of the most critical global issues, exacerbated by rapid industrialization and urbanization in the twenty-first century. Among various pollutants, phenolic compounds are particularly concerning due to their high toxicity, persistence in the environment, and adverse effects on human and ecosystem health (Dhatwalia and Nanda 2019). Phenols are commonly found in petrochemical industry waste and have the potential to disrupt biological wastewater treatment processes by inhibiting microbial activity (Morones-Esquivel et al. 2022). Due to their carcinogenic, teratogenic, and mutagenic properties, organizations such as the U.S. Environmental Protection Agency (EPA) and the European Union classify phenols as priority pollutants that require strict removal standards (U.S. Environmental Protection Agency (EPA). 2002).

Traditional methods for removing phenolic compounds include chemical oxidation, solvent extraction, and adsorption. However, these methods face challenges such as high operational costs and the formation of secondary pollutants (Dhatwalia and Nanda 2019). In contrast, biodegradation offers a more sustainable and cost-effective alternative. Microorganisms capable of degrading phenolic compounds can completely mineralize these compounds by utilizing them as a carbon and energy source. Research has identified several microbial species with high phenol degradation potential, including *Pseudomonas putida* and *Rhodococcus opacus* (Kumar et al. 2005; Anokhina et al. 2025).

Recent studies further highlight the effectiveness of specific bacterial strains and consortia in degrading high concentrations of phenol. For example, a bacterial consortium of *Pseudomonas stutzeri* N_2_ and *Rhodococcus qingshengii* FF completely degraded 2450 mg/L phenol within 36 h while simultaneously removing 97.08% of petroleum hydrocarbons from semi-coking wastewater (Bai et al. 2022). Similarly, *Rhodococcus pyridinivorans* PDB9T N-1 achieved complete degradation of 1600 mg/L phenol in 56 h and removed over 97.5% TOC at 1000 mg/L (Barik et al. 2021). In another study, *Pseudomonas aeruginosa* and *Klebsiella variicola*, isolated from sewage sludge, degraded over 70% of 1000 mg/L phenol within three days and significantly reduced phenol toxicity based on soybean germination tests (Mahgoub et al. 2023).

Advancements in molecular biology and genomics have significantly improved our understanding of metabolic pathways involved in phenol degradation (Xu et al. 2021; Viggor et al. 2020). The identification of genes and enzymes responsible for the breakdown of phenolic compounds has enabled the development of genetically engineered microorganisms with enhanced degradation capabilities (Anokhina et al. 2025; Rucká et al. 2017). For instance, expression analyses in phenol-degrading bacteria have confirmed the activation of meta- and ortho-cleavage pathway genes, contributing to efficient aromatic ring breakdown (Mahgoub et al. 2023). Additionally, bioaugmentation strategies, which involve the introduction of specialized microbial cultures into contaminated environments, show promise for accelerating the degradation process (Bai et al. 2022; Oro et al. 2024).

Despite these advances, challenges remain in optimizing microbial degradation processes for industrial applications. Factors such as the presence of co-contaminants, variability in wastewater composition, and fluctuating environmental conditions can significantly impact biodegradation efficiency (Tesfaye et al. 2025). Studies on aerobic granular sludge cultivated in sequencing batch reactors, as well as acclimated microbial consortia adapted to high phenol loads, demonstrate that well-structured microbial communities can withstand phenolic stress and efficiently treat phenol-containing wastewaters. (Tomar and Chakraborty 2020; Prpich and Daugulis 2005). However, comparatively limited information is available on the phenol and mixed phenolic compound degradation performance of non-model environmental bacterial isolates, particularly under adaptive exposure and conditions that better reflect the complexity of industrial wastewater. Recent studies have highlighted the challenges associated with complex wastewater matrices containing diverse organic pollutants, such as those encountered in landfill leachate and other industrial effluents (Gaur et al. 2024), further motivating the present investigation into microbial degradation under environmentally relevant mixed-pollutant conditions.

Although phenol biodegradation has been extensively documented across diverse bacterial taxa, species-specific evidence within certain genera remains limited. Several environmental *Microbacterium* spp. have been detected in phenolic and lignocellulosic pollutant–contaminated environments, and members of the genus are frequently reported within aromatic-compound-degrading consortia (Farías et al. 2022). Similarly, *Brevundimonas* spp. have been identified as participants in mixed microbial systems capable of transforming phenolic and related organic contaminants (Farías et al. 2022). Broader reviews on bacterial phenol degradation indicate that many environmental isolates contribute to the breakdown of phenol through diverse metabolic pathways (Adetitun and Tomilayo 2024; Krastanov et al. 2013). Moreover, phenol-degrading bacteria isolated from contaminated soils and wastewaters, such as *P. aeruginosa* and *K. variicola*, demonstrate that non-model environmental isolates can exhibit strong phenol degradation capacity under stress conditions (Mahgoub et al. 2023). However, despite the increasing documentation of phenol-degrading consortia and genera, no prior study has explicitly demonstrated phenol degradation by *Microbacterium arabinogalactanolyticum* or *Brevundimonas diminuta* in pure culture, nor examined their performance under mixed phenolic loads or adaptive-enrichment conditions relevant to industrial wastewater.

In this context, the objectives of the present study were to investigate the phenol and mixed phenolic compound degradation potential of two environmental bacterial isolates, *M. arabinogalactanolyticum* and *B. diminuta*, obtained from industrially impacted environments. Although phenol biodegradation has been extensively studied using model organisms, information regarding the degradation performance of these species, particularly under mixed phenolic loads and following adaptive exposure, remains limited. The novelty of this work lies in (i) the isolation and adaptive enhancement of phenol-tolerant strains from refinery-associated sources, (ii) the comparative evaluation of single-strain and defined consortium-based degradation under environmentally relevant phenolic mixtures, and (iii) the assessment of degradation kinetics relevant to bioaugmentation strategies for industrial wastewater treatment.

## Materials and methods

### Sample collection

The microorganisms used in this study were isolated from the activated sludge unit of a petroleum refinery wastewater treatment plant and soil contaminated with petroleum hydrocarbons near a bus terminal in İzmir. Samples were transported at 4 °C in sterile containers and processed within 24 h to preserve microbial viability.

### Isolation and enrichment of microorganisms capable of phenol and phenolic compound degradation

Microorganisms capable of degrading phenol and its derivatives were enriched in 500 mL Erlenmeyer flasks containing 100 mL Bushnell Haas Yeast Broth (BHY), prepared with 0.327 g Bushnell Haas Broth and 0.1 g yeast extract. Phenol (20 mg/L) and a mixture of phenolic compounds—including o-cresol, p-cresol, m-cresol, 2,3-dichlorophenol, 2,4-dichlorophenol, 3,4-dichlorophenol, 2,4-dinitrophenol, and 2,5-dichlorophenol—were added at a combined concentration of 30 mg/L as the sole carbon sources. Each flask was inoculated with 5 mL of activated sludge and incubated at 27 °C with shaking at 150 rpm for 5 days.

The initial concentrations of phenol and mixed phenolic compounds were selected to reflect environmentally relevant levels commonly reported in refinery wastewater influents, while avoiding acute inhibitory effects on microbial activity. Previous studies indicate that phenol concentrations in petroleum refinery wastewaters may range from low mg/L levels up to several hundred mg/L, with concentrations between 10 and 100 mg/L frequently encountered at the inlet of biological treatment units. Accordingly, an initial phenol concentration of 20 mg/L and a total mixed phenolic concentration of 30 mg/L were chosen to simulate realistic, sub-inhibitory conditions suitable for microbial enrichment and adaptive selection.

The phenolic mixture was composed of cresols, chlorophenols, and 2,4-dinitrophenol, which are among the most frequently detected phenolic derivatives in refinery and petrochemical effluents. These compounds represent different degrees of molecular complexity and toxicity, allowing evaluation of microbial degradation performance under chemically diverse yet environmentally relevant conditions. Sequential passaging with gradually increasing concentrations was subsequently applied to enhance phenol tolerance and to simulate fluctuating phenolic loads characteristic of industrial wastewater systems.

Rather than focusing on high concentrations of individual compounds, the selected mixture and combined concentration were designed to reflect the co-occurrence of multiple phenolic derivatives at low to moderate levels, which is a typical characteristic of refinery wastewater streams prior to biological treatment. This approach enables a more realistic assessment of microbial degradation behavior under mixed-pollutant conditions relevant to industrial wastewater treatment systems.

### Sequential passaging and tolerance enhancement

At the end of the five-day incubation period, 2.5 mL samples were taken from each culture and transferred to new 250 mL Erlenmeyer flasks containing 50 mL of fresh BHY medium. The concentrations of phenol and phenolic compounds in the medium were gradually increased over three passages. By the third passage, the medium was prepared to contain 50 mg/L phenol and 100 mg/L phenolic compounds. All samples were incubated under the same conditions.

Following sequential passaging, cultures were serially diluted (10^−1^–10^−4^) and spread onto Tryptic Soy Agar (TSA) plates. Plates were incubated at 27 °C for 48 h. Colonies exhibiting distinct morphological characteristics were purified using the streak plate method. Purified isolates were examined under a light microscope to assess cell shape and motility.

### Molecular characterization of isolates

Genomic DNA was extracted from the most efficient degrading isolates using the ZR Fungal/Bacterial DNA MiniPrep Kit (Zymo Research, USA). Total genomic DNA (25–50 ng) was used as a template for amplifying the 16S ribosomal RNA (16S rRNA) coding region using universal primers 11F (5’- GTTTGATCCTGGCTCAG-3’) and 1492R (5’-TAC GGC TAC GAC TT-3’) (Zhang et al. 2015).

PCR amplification was performed using a TechnePlus Thermal Cycler under the following conditions: initial denaturation at 95 °C for 5 min, followed by 30 cycles of denaturation at 95 °C for 20 s, annealing at 48 °C for 30 s, and extension at 72 °C for 90 s, with a final extension at 72 °C for 5 min.

PCR products were visualized by electrophoresis on a 1% agarose gel using a multispectral imaging system (BioSpectrum, UK). The 1480 bp PCR fragments were purified using the NucleoSpin Gel and PCR Clean-up Kit (Macherey–Nagel GmBH, Germany) and sequenced using an automated ABI Prism Genetic Analyzer (Applied Biosystems, USA). The sequences were edited using DNA Baser Sequence Assembler software and analyzed using the BLAST platform in the National Center for Biotechnology Information (NCBI). Multiple sequence alignment was performed with MEGA 7 software. The evolutionary history was traced using the Neighbor-Joining and Maximum Likelihood methods (Tamura et al. 2011, 2004; Kumar et al. 2018).

### Evaluation of the biodegradation efficiency of isolated microorganisms

Sequentially passaged isolates were cultured in 50 mL of nutrient broth and incubated at 27 °C for 24 h in a shaking incubator at 150 rpm. After incubation, 1 mL of each culture was transferred to sterile centrifuge tubes and centrifuged at 3000 rpm for 5 min. The supernatant was discarded to remove residual organic matter, and the microbial pellets were resuspended in 1 mL sterile water. This washing step was repeated three times, and the final suspension was used for inoculation.

Biodegradation experiments were conducted in 250 mL sterile Erlenmeyer flasks containing 50 mL BHY medium. The isolates were inoculated to achieve an initial optical density (OD600) of 0.1. Control flasks without inoculum were included to monitor abiotic degradation.

#### Experimental design

## Stage 1: Pure phenol degradation:

Each isolate was cultured in BHY medium containing 50 mg/L phenol. Samples were collected every 6 h for 36 h. Phenol concentration was measured using HPLC, and OD600 values were recorded to generate growth curves.

## Stage 2: Mixed phenolic compounds:

Isolates were incubated in BHY medium containing 20 mg/L phenol and 30 mg/L total phenolic compounds for 120 h. Samples were collected every 6 h and analyzed by HPLC. Experiments were conducted separately for each isolate.

## Stage 3: Mixed culture degradation:

Equal volumes of each isolate were combined and inoculated into BHY medium containing 20 mg/L phenol and 30 mg/L phenolic compounds. The final OD600 was adjusted to 0.1. Samples were collected periodically over 120 h and analyzed by HPLC.

### HPLC analyses

Phenol and phenolic compound concentrations were quantified using high-performance liquid chromatography (HPLC). Prior to analysis, samples were filtered through 0.22 µm membrane filters. An Agilent 1100 Series HPLC system equipped with a C18 column (30 °C) and a diode array detector (DAD) was used. The flow rate was set to 0.8 mL/min.

**Mobile phase:**

- Acetic acid–water (5:95) for phenol, 2,4-dinitrophenol, and cresols.
- Methanol–acetic acid (5:95) for chlorophenols.

Standard calibration curves were generated for each compound (R^2^ > 0.995). Limits of detection (LOD) and quantification (LOQ) were determined. All analyses were performed in triplicate. Degradation efficiency was calculated as the percentage decrease in compound concentration over time.

## Results

### Microbial isolation and phylogenetic analysis

Among 15 isolates obtained from refinery wastewater and contaminated soil, two strains—designated VGT4 and PKN7—exhibited the highest phenol and phenolic compound degradation capacities following sequential passaging.

**Fig. 1:**
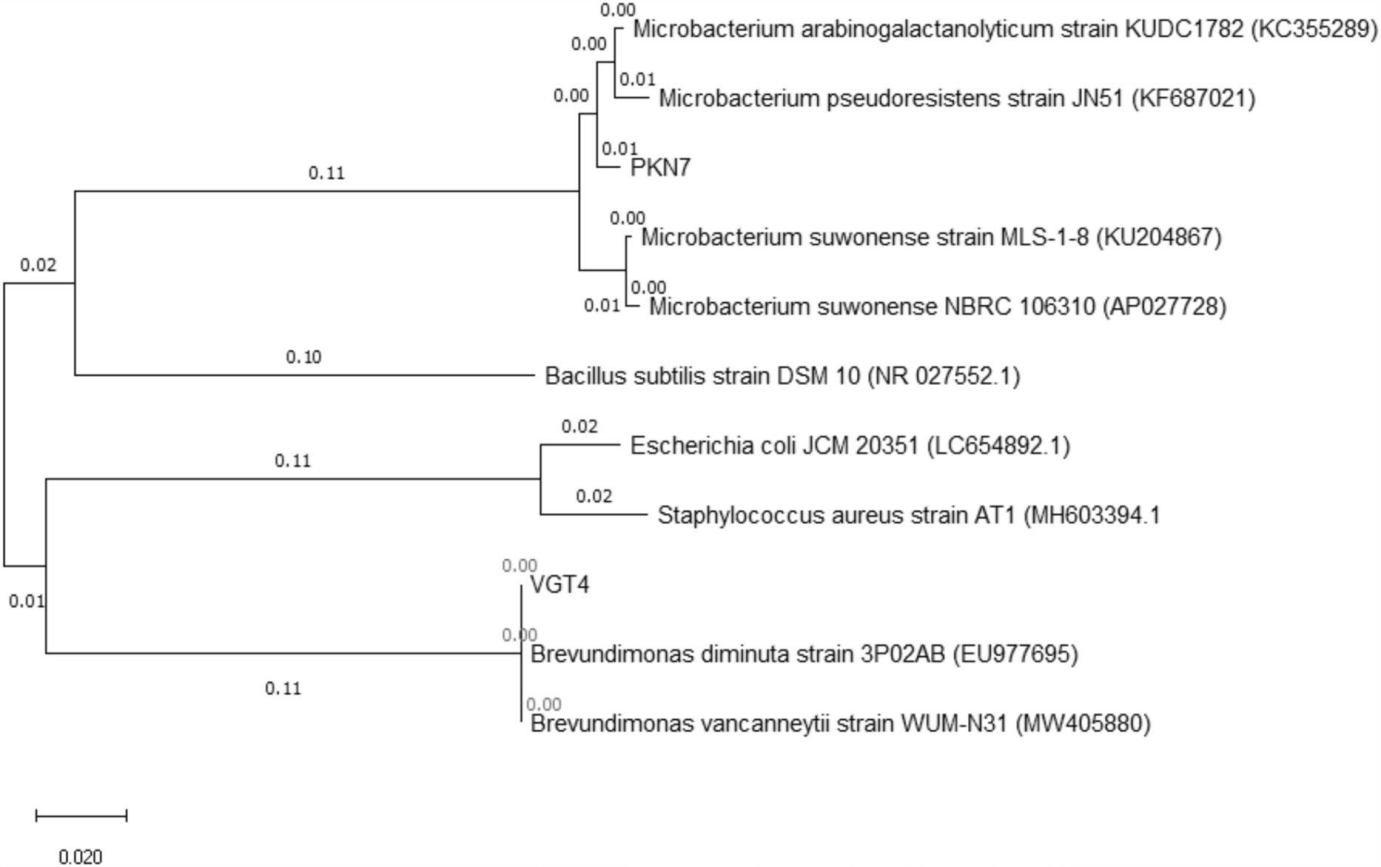
Phylogenetic tree based on 16S rRNA gene sequences showing the position of isolates VGT4 and PKN7 among closely related reference species. The evolutionary history was inferred using the Neighbor-Joining method with 1000 bootstrap replicates. Scale bar represents the number of substitutions per site

Morphological and cultural characteristics of these isolates are summarized in Table 1. Sequence-based identification using BLAST analysis revealed that isolate PKN7 showed the highest similarity to *Microbacterium arabinogalactanolyticum*, with a percentage identity of 98.55% and 100% query coverage (GenBank accession number: MW547434). Similarly, isolate VGT4 exhibited 98.85% sequence identity and 100% query coverage with *Brevundimonas diminuta* (GenBank accession number: MW547436). These results support species-level identification based on 16S rRNA gene sequence similarity (Fig. 1). Phylogenetic analysis based on the 16S rRNA gene sequences showed that both isolates clustered consistently with their respective reference species. The branching patterns supporting the placement of isolate PKN7 within the *M. arabinogalactanolyticum* clade and isolate VGT4 within the *B. diminuta* clade were supported by high bootstrap values obtained from 1000 replicate analyses, indicating robust phylogenetic affiliation and reliable strain placement (Fig. 1).

**Table 1:** Morphological characteristics of the isolates

FEATURE	PKN7	VGT4
Cell morphology	Rod	Rod
Colony pigmentation	White	Yellow
Gram staining	+	−
Optimum temperature (°C)	27	27

### Degradation activities of phenol and phenolic compounds

**Fig. 2:**
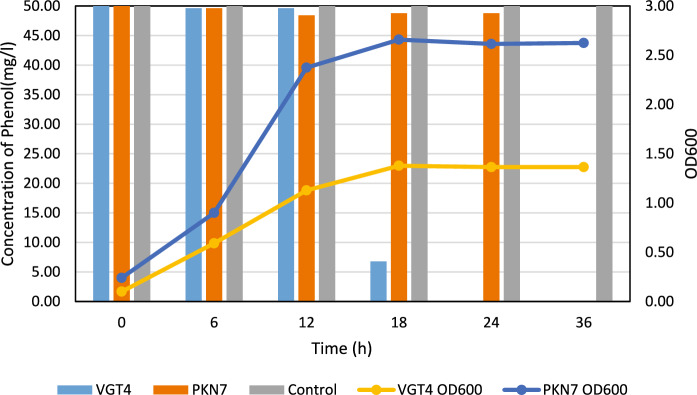
Growth curve (OD600) and phenol degradation profile of isolates VGT4 and PKN7 in Bushnell Haas Yeast medium containing 50 mg/L phenol. Data are presented as mean ± standard deviation (n = 3)

As shown in Fig. 2, HPLC analyses indicate that VGT4 completely removed phenol by the 24th hour, whereas PKN7 achieved complete degradation by the 36th hour. During this period, VGT4 exhibited a higher growth rate, as reflected in OD600 measurements. Data are presented as mean ± standard deviation (n = 3).

**Fig. 3:**
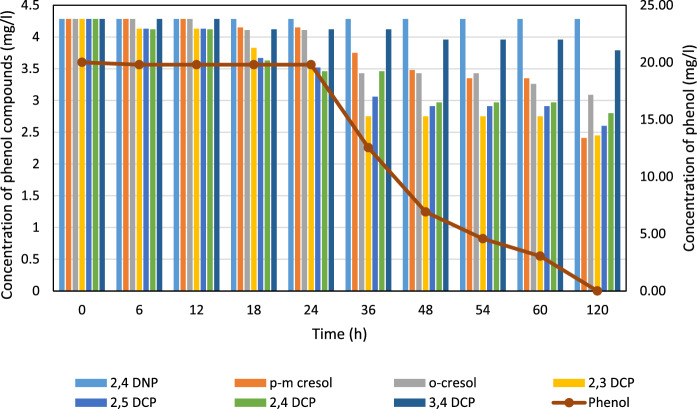
Degradation of phenol and phenolic compounds by isolate VGT4. Concentrations were monitored by HPLC over 120 h. Data are presented as mean ± standard deviation (n = 3)

**Fig. 4:**
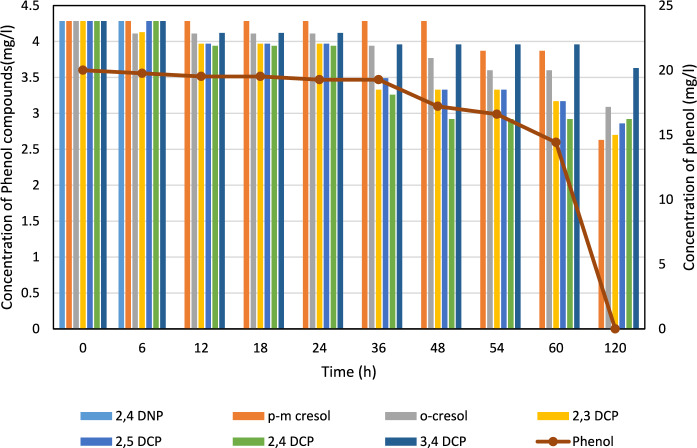
Degradation of phenol and phenolic compounds by isolate PKN7. Phenol was completely degraded within 120 h, while partial degradation of chlorophenols and cresols was observed. Data are presented as mean ± standard deviation (n = 3)

Figures 3 and 4 illustrate the degradation profiles of phenol and phenolic compounds by isolates VGT4 and PKN7, respectively. According to Fig. 3, VGT4 achieved complete degradation of phenol by 120 h but showed limited degradation of other phenolic compounds. In contrast, Fig. 4 shows that PKN7 initiated degradation of 2,4-dinitrophenol after 6 h and fully degraded phenol by 120 h, with partial degradation of other phenolic compounds.

**Fig. 5:**
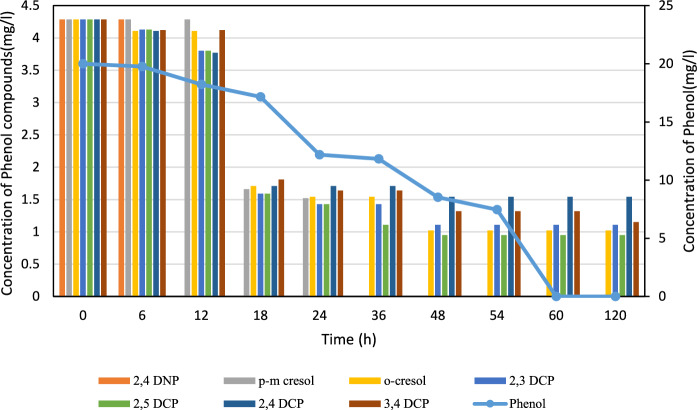
Degradation of phenol and phenolic compounds by the mixed culture of isolates VGT4 and PKN7. The consortium achieved complete degradation of 2,4-dinitrophenol within 12 h and phenol within 60 h. Other phenolic compounds showed degradation efficiencies ranging from 73.16 to 77.82%. Data are presented as mean ± standard deviation (n = 3)

Figure 5 presents the degradation performance of the mixed culture of VGT4 and PKN7. The consortium completely degraded 2,4-dinitrophenol within 12 h and phenol within 60 h. Other phenolic compounds were degraded with efficiencies ranging from 73.16 to 77.82%**.**

Sequential passaging enhanced the tolerance and degradation efficiency of the isolates, indicating adaptive responses suitable for dynamic industrial environments. These findings demonstrate the potential of *M. arabinogalactanolyticum* and *B. diminuta* as robust candidates for the biological treatment of phenolic pollutants in industrial wastewater.

## Discussion

This study demonstrates that the isolates VGT4 and PKN7 possess strong potential for the biodegradation of phenolic compounds. The synergistic effect observed in mixed culture experiments (Fig. 5) is consistent with the advantages of microbial consortia reported by Perpetuo et al. (2012). In addition, the ability of the isolates to degrade different phenolic derivatives supports the importance of microbial diversity in bioreactor applications, as highlighted by Arutchelvan et al. (2006). These findings emphasize the adaptive capacities of the isolates and their potential for treating phenolic pollutants in complex wastewater matrices.

HPLC analyses confirmed that *M. arabinogalactanolyticum* and *B. diminuta* exhibited complete phenol removal and high degradation efficiency under controlled laboratory conditions at 27 °C. Notably, *M. arabinogalactanolyticum* also degraded 2,4-dinitrophenol, a compound generally considered recalcitrant. Although phenol-degrading bacteria have been widely reported across several genera (Mahgoub et al. 2023; Adetitun and Tomilayo 2024; Krastanov et al. 2013), *to the best of our knowledge*, there are no published reports describing phenol degradation by *M. arabinogalactanolyticum* or *B. diminuta* in pure culture. This highlights the species-level novelty of the present work and reinforces the relevance of these isolates as alternative, non-model candidates for phenolic wastewater treatment. Their ability to degrade mixed phenolic loads following adaptive exposure further suggests that these strains possess metabolic flexibility beyond what is typically reported for environmental isolates (Farías et al. 2022).

It should be noted that degradation efficiency in this study was assessed based on time-dependent HPLC disappearance of the parent phenolic compounds. Although compound removal, coupled with cell growth and negligible loss in abiotic controls, strongly suggests biologically driven degradation rather than adsorption, HPLC disappearance alone does not confirm complete mineralization, as intermediate metabolites may still be (Adetitun and Tomilayo 2024; Krastanov et al. 2013). Phenolic pollutants often undergo stepwise transformation through monooxygenation, deamination, or ring-cleavage pathways, and identification of downstream metabolites is required to differentiate partial degradation from full mineralization (Mahgoub et al. 2023). Therefore, the results reported here should be interpreted as biodegradation capacity rather than confirmed mineralization. Future studies using LC–MS or GC–MS metabolite profiling, along with TOC/COD analyses, will be essential to resolve metabolic pathways and determine whether complete mineralization occurs.

The degradation profiles obtained in this study are comparable to those reported for halophilic and phenol-tolerant bacterial strains by Arutchelvan et al. (2006), confirming the resilience and adaptability of the isolates. The sequential passaging approach clearly enhanced phenol tolerance, indicating that the strains can physiologically adapt to elevated phenolic loads—an important characteristic for real industrial wastewater treatment systems where influent phenol concentrations may fluctuate significantly over time.

### Comparative analysis of mixed and pure cultures

Mixed culture experiments revealed enhanced degradation performance compared to pure cultures, indicating cooperative interactions between the isolates. For instance, 2,4-dinitrophenol was degraded within 12 h in mixed culture, compared with 36 h in pure cultures. These findings are consistent with previous studies demonstrating that microbial consortia often outperform individual strains due to synergistic metabolic interactions, cross-feeding, or sequential substrate utilization (Perpetuo et al. 2012). Although synergistic metabolism is likely a key driver of the observed enhancement, alternative explanations—including differences in growth kinetics, substrate affinity, or stress tolerance—may also contribute to the improved performance observed in mixed cultures.

### Implications for industrial applications

The ability of these isolates to efficiently remove and degrade phenolic compounds under laboratory conditions suggests their potential as promising candidates for industrial wastewater treatment. Their adaptability to multiple phenolic derivatives, combined with the enhanced performance observed in mixed culture, supports the use of defined microbial consortia as a sustainable and cost-effective alternative to conventional physicochemical treatment methods. These findings also contribute to the growing body of evidence supporting bioaugmentation strategies, underscoring the importance of community-level interactions for optimizing phenolic pollutant removal in refinery wastewater and other industrial effluents.

However, the present findings are based on laboratory-scale experiments, and further validation using real industrial wastewater and pilot-scale systems will be required to assess long-term stability and field applicability. In particular, evaluating performance under varying hydraulic retention times, shock phenol loads, salinity fluctuations, and nutrient limitations will be critical for understanding operational robustness in real-world treatment settings.

## Conclusion

This study successfully isolated and characterized two bacterial strains, *M. arabinogalactanolyticum* and *B. diminuta*, that demonstrated complete phenol degradation under laboratory conditions. Their combination in mixed cultures accelerated the breakdown of complex phenolic mixtures, highlighting the benefit of synergistic interactions.

These findings establish the novelty of the identified strains and provide baseline evidence for their use in bioremediation. Future work should focus on validating their performance under variable environmental conditions and scaling up the process in pilot-scale bioreactors.

## Study limitations

This study was conducted under laboratory-scale conditions using synthetic media and controlled parameters. Therefore, the degradation performance observed here may differ under real refinery wastewater matrices that contain complex co-contaminants, fluctuating loads, and operational variability. In addition, biodegradation was primarily assessed based on time-dependent disappearance of parent phenolic compounds by HPLC; metabolite identification and pathway-level confirmation were not performed. Future work should validate the performance of these isolates/consortium in real wastewater and pilot-scale systems, supported by metabolite profiling (e.g., LC–MS/GC–MS) and complementary bulk parameters such as TOC/COD to better assess transformation and mineralization.

## Data Availability

The authors conﬁrm that the data supporting the ﬁndings of this study are available within the article.
